# Community perceptions about dementia in southwestern Uganda

**DOI:** 10.1186/s12877-020-01543-6

**Published:** 2020-04-15

**Authors:** Judith Owokuhaisa, Godfrey Zari Rukundo, Edith Wakida, Celestino Obua, Stephanie S. Buss

**Affiliations:** 1 Department of Internal Medicine, Faculty of Medicine, Mbarara University of Science and Technology, P. O. Box 1410, Mbarara, Uganda; 2 Department of Psychiatry, Faculty of Medicine, Mbarara University of Science and Technology, Mbarara, Uganda; 3 Office of Research Administration, Faculty of Medicine, Mbarara University of Science and Technology, Mbarara, Uganda; 4 Department of Pharmacology, Faculty of Medicine, Mbarara University of Science and Technology, Mbarara, Uganda; 5 Berenson-Allen Center for Noninvasive Brain Stimulation, Division of Cognitive Neurology, Department of Neurology, Beth Israel Deaconess Medical Center, Harvard Medical School, Boston, MA USA

**Keywords:** Dementia, Culture, Social determinants of health, Social perception, Community mental health services, Uganda, Global health

## Abstract

**Background:**

With the increasing number of people surviving into older age in Africa, dementia is becoming a public health concern. Understanding the social dynamics of dementia in resource-limited settings is critical for developing effective interventions. We explored community perceptions about people with dementia in southwestern Uganda.

**Methods:**

Fifty-nine individuals (aged 19–85 years, 56% female) participated in seven focus group discussions. In addition, 22 individual in-depth interviews were conducted among individuals (aged 22–84 years, 36% female). Both interviews and focus group discussions were audio recorded, transcribed verbatim, and evaluated using a quantitative content analysis approach.

**Results:**

Five themes were generated during content analysis: i) Labeling of the illness, ii) Presentation of the person with dementia, iii) Causation, iv) Impact of the disease on people with dementia and their caregivers and v) Views on how to address unmet needs in dementia care. Dementia was commonly referred to as “*okuhuga****”****or “okwebwayebwa” (also, oruhuzyo/ empugye / akahuriko)* which translates as “mental disorientation”. The participants reported that most people with dementia presented with forgetfulness, defecating and urinating on themselves, wandering away from home, going out naked, and picking up garbage. Some participants perceived memory problems as a normal part of the aging process, while others attributed the cause of dementia to syphilis, cancer, allergy, old age, satanic powers, witchcraft, poor nutrition, or life stress. Participants reported multiple sources of stress for caregivers of people with dementia, including financial, social, and emotional burdens. Finally, participants suggested that community and governmental organizations should be involved in meeting the needs of people with dementia and their caregivers.

**Conclusions:**

Community members in southwestern Uganda largely identified dementia as a problem that comes with older age, and can identify key features of dementia presentation. Participants identified significant stressors affecting people with dementia and their caregivers, and reported that families and caregivers would benefit from education on the management of symptoms of dementia, and assistance in overcoming associated financial, social, and emotional burdens related to caretaking.

## Background

Worldwide, there are estimated to be around 50 million people living with dementia, and this number is expected to triple to 115.4 million by 2050 [1]. The largest increase in dementia burden is projected to occur in low and middle income countries (LMIC) such as Uganda [2].

People with dementia (PWD) gradually lose cognitive function, resulting in an inability to perform activities of daily living (ADLs) [3]. A study of hospitalized patients in Uganda over the age of 60 found that 5.5% were diagnosed with dementia, but 14% had evidence of cognitive impairment on the MMSE (score < 19), suggesting that the true prevalence of dementia may already be higher than reported [4]. Therefore the provision of effective services to the increasing numbers of individuals with dementia is a public health care priority [3]. Early and timely diagnosis is paramount to providing individuals with access to appropriate medical care and support services [5]. Interventions such as memory clinics, geriatric services, social work, and caregiver support programs assist in the diagnosis and management for PWD [5]. However, in LMIC there is limited human and technological capacity to address the increasing demand of chronic diseases [6], so most of the burden of dementia care falls on caregivers and local communities [7]. There are several factors affecting the health-seeking behaviors of PWD and their caregivers, including perceptions about the disease and socio-cultural factors influencing formal and informal caregiving for PWD [8]. Understanding the community perceptions of dementia will assist healthcare policy makers to design new interventions to help PWD and their caregivers in LMIC.

Prior studies of community perceptions of dementia in Sub-Saharan Africa show that the cause of dementia is attributed to old age, spiritual beliefs, and fate from earlier wrong doing [9], or to witchcraft [10, 11]. These perceptions have been known to lead to fear, stigma, discrimination, and social isolation of PWD; and in some cases even violence or murder [11–13] . Little work has been done in Uganda to characterize community perceptions about dementia, and there is paucity of information about the impact of dementia on PWD and caregivers. The aim of this study was to identify community perceptions about dementia in southwestern Uganda using focus-group discussions (FGDs) and individual in-depth interviews (IDIs) with local community members.

## Methods

### Study design

This was across-sectional qualitative study that used thematic analysis to identify community perceptions about dementia in southwestern Uganda. We used semi-structured interview guides for FGDs and IDIs. Interview questions were designed based on the Explanatory Model Framework to assess how individuals name the illness, describe it’s causality and meaning, and understand the effects and treatments of disease [14]. The explanatory model allows researchers to assess how individuals communicate when discussing issues related to health and illness [15].

### Study setting

The study was conducted in three villages within separate districts in southwestern Uganda: Nyakabungo in Kabale, Kasharira in Ntungamo, and Nyakakoni in Mbarara districts. These districts were chosen because they had a substantial older population, with the number of people aged 60 and above numbering 29,672 in Kabale, 23,344 in Ntungamo, and 20,442 in Mbarara. People from these districts practice crop and animal farming and identify with several different tribes including Banyankore, Bakiga, Bafumbira, Bahiima, and Banyarwanda.

### Participants’ recruitment

IDIs were conducted with (i) Traditional healers, who deal with many of the mental health care needs of the community in Uganda [7]; (ii) Local council leaders, who serve as political, civic, and opinion leaders in the community; (iii) Community elders, who are respected individuals in the community, regarded as having a good knowledge of their culture, and serve as a source of wisdom and advice; (iv) Religious leaders, who play part in lay mental health services in the community; and (v) Community development officers, who are responsible for the social and economic development in the community. Participants who took part in the FGDs included a diverse array of local community members, council leaders, religious leaders, community elders, traditional healers, and community development officers. Participants were recruited through purposive sampling to ensure that the broadest range of information and perspectives was obtained. Age and gender guided the purposive sampling technique. Recruitment was stopped when saturation level and redundancy were reached. FGDs were stratified by gender.

### Procedures

In-depth interviews and FGDs were conducted by the lead author (JO) and two trained research assistants in December 2018. Interviews lasted approximately 30–70 min in free and flowing discussions were audio taped. Participants were asked questions in “Runyankore – Rukiga” which is the local language spoken by participants. Audio interviews were transcribed verbatim in the local language and then those transcripts were translated into English by research assistants with supervision from JO. This process was chosen to ensure the original meaning of participants’ statements so that responses was not altered or lost.

### Data collection and tools

A semi-structured interview guide was developed by JO in consultation with GZR, CO, EW and SB. Questions were developed based on the explanatory model for understanding cultural perceptions about illness. The interviewer used semi-structured interviews in order to probe for clarity and to follow leads that were brought up by the participants. The interview guides were first pilot tested in a community that was not part of the study.

For both the FDGs and IDIs, the discussion started with an introduction in which either the moderator or first author JO explained the purpose of the research. The opening question was: “What are the major health problems encountered by elderly people in this community?” If forgetfulness or memory loss was not mentioned among the health problems, it was inquired about. The, participants were then asked further questions about memory loss in older people: “What is it called?” “How does it present?” “What causes the illness?” “What effect does it have on the patient and family?” and “How it is usually treated?”

### Data management and analysis

Data were transcribed verbatim by the research assistants, and checked by JO against the audio recordings for correctness of information. The first set of transcripts was reviewed by GZR to ensure that the data collection and methodology were accurate. Data were thematically analyzed [16] with the help of the Atlas.ti version 7 software. The initial coding was done by JO, after which the data were reviewed and reorganized into categories in order to search for systematic relationships. The five a priori themes were modified and additional subthemes were identified based on the common patterns that emerged from the aggregated data: labeling, causality, presentation, effect, and treatment of dementia. Coding was performed by comparing statements among participants and searching for regularities, patterns, and contradictions.

## Results

A total of 22 IDIs and seven FGDs were conducted for our study. Seven IDIs and two FGDs were from Kabale, nine IDIs and two FGDs from Ntungamo, and six IDIs and three FGDs from Mbarara districts. A total of 59 participants (26 men and 33 women) participated in FGDs. Participants were aged between 19 and 85 years. Table 1 shows a summary of in-depth interview participants. Table 2 shows the five major themes that emerged from the aggregated data: (a) Labeling the illness, (b) Presentation of person with dementia, (c) Causation, (d) Impact of the disease, and (e) Views on how to address unmet needs in dementia care.

**Table 1:** Summary of in-depth interview participants characteristics

***Type of respondent***	***Total number of participants by gender***	***Age Range (years)***
***Traditional healer***	***Male (n = 0)***	***–***
	***Female (n = 2)***	***52–66***
***Local council leader***	***Male (n = 5)***	***27–60***
	***Female (n = 0)***	***–***
***Community elder***	***Male (n = 1)***	***72***
	***Female (n = 2)***	***66–84***
***Religious leader***	***Male (n = 8)***	***30–74***
	***Female (n = 2)***	***51–70***
***Community development officer***	***Male (n = 0)***	***–***
	***Female (n = 2)***	***24–25***

Legend: Characteristics of participants from in-depth interviews were shown, divided by category of local leader, gender, and age range

**Table 2:** Summary of themes and sub themes

***Theme***	***Sub theme***	***Category***
1) *Labeling the illness*		
* 2) Presentation of a person with dementia*		
3) *Causation*	*Biological, aging, psychological, nutritional deficiencies and substance abuse, poverty, social and traditional beliefs*	
4) *Impact of the disease*	*Impact of the disease on PWD* *Impact of the disease on the caregiver*	*Inability to perform personal care, risk of medical and psychological illness, mistreatment and risk of elder abuse* *Financial stress, social isolation, handling problematic behaviors, physical and emotional stress*
5) *Views on how to address unmet needs in dementia care*	*Emotional support* *socioeconomic support* *cultural sensitization*	

Legend: A summary of themes, sub themes and categories of community perceptions about dementia in southwestern Uganda

### Theme 1: labeling the illness

In our study, participants described dementia as a feature of old age, the most common label was “akuzire” which meant “old age”. Other labels that emerged from the data included *awusse* (“rotten in the head”), *atabukiire*/o*kuhungutuka* (“mad” or “mentally disturbed”), *tayiine obwengye* (“lacks knowledge”), *ebitekateko bikuzire* (“old thoughts”), *nayebwayebwa* (‘so forgetful’), *bukuru/akuziire/empinduka ya bukuru* (“old age”), and *omwaga/oburomborombo* (“irritability”).

*“In our community when someone becomes forgetful people do not know or tell that it is a disease, they start saying that elderly man is mentally disoriented so they look at him as useless person (IDI Male)”.*

*“We call it old age, when a person becomes elderly his knowledge reduces and he starts forgetting (IDI Female)”.*

When participants were asked to elaborate on the difference between the various labels given to dementia there was an indication that all these labels could be used interchangeably to show a relationship between forgetfulness among elderly persons.

### Theme 2: presentation of person with dementia

Participants identified cognitive symptoms such as memory loss as well as behavioral changes associated with dementia. Participants reported that most PWD present with forgetfulness, defecate and urinate on themselves, play with waste, go outside naked, or pick up garbage.

*“When she has memory loss you cloth her or make her bed she undresses and throws [the clothes] away. Sometimes she urinates and smears herself with urine”. (IDI Female)*

*“You may wake up and bathe her she says that you have not … when she urinates she argues that [she has been rained on] … you bring for her food and she says that the plate is dirty … she defecates and starts to play with her waste. That is when you know that she has memory loss” (FGD Female).*

Responses are summarized in Table 3. There was no difference in responses based on gender between IDIs and FGDs.

**Table 3:** Symptoms of dementia as reported by participants

*“Forget the road”*	
*“Forget people, including their own children”*	
*“Forget to eat or to change clothes”*	
*“Memory loss”*	
*“Behaves like a child”*	
*“Uncoordinated conversation”*	
*“Picking empty bottles”*	
*“Going naked”*	
*“Wandering”*	
*“Putting on torn clothes”*	
*“Sleeplessness”*	
*“Suicidal ideation”*	
*“Sits anywhere”*	
*“Crying/ wailing”*	
*“Defecates anywhere and on self”*	
*“Eats goats feces”*	
*“Scatolia (Smearing of feces)”*	
*“Become mad”*	

Legend: Participants described a variety of signs and symptoms of dementia; the most common responses are summarized

### Theme 3: causation

In relation to causation, participants in our study identified multiple causes as contributing to dementia. These included biological, psychological, and social causes. Participants frequently combined explanations relating to both the proximate (how) and the ultimate (why) causes of memory loss. In this study, the causes were grouped under seven sub themes: (i) Biological, (ii) Aging, (iii) Psychological, (iv) Nutritional deficiencies and substance abuse, (v) Poverty, (vi) Social, and (vii) Traditional beliefs.

#### Biological

Some participants attributed forgetfulness among the elderly to medical conditions such as syphilis (*ebinyoro*), cancer (*ekokoro*), and allergy (*efumbi*). Some participants believed that prolonged illness coupled with taking multiple medications was the cause of dementia.

*“Another thing which causes forgetfulness among the elderly, there is when you find that a person has suffered from a disease for a long time like cancer and he takes different types of medicine so it brings him memory loss”.(IDI Male)*

Majority of the participants related dementia to aging and thinking too much following loss of property or children. But terms like degenerative disorder or disorder of the brain were not specifically used by the participants.

#### Aging

Some participants insisted that the only cause of dementia was advanced age. This was accompanied by a belief that memory loss was normal in the elderly, and that aging leads to physical changes which cause physical weakness and mental changes leading to the inability to perform personal activities.

*“I think its old age because old age takes everything, when you become elderly you get disconnected”.(IDI Female)*

*“For me for, a person to become elderly and become forgetful, I think that its old age.” (FGDFemale)*

*“So, we accept that it is age and it has no cure”. (FGD Female)*

#### Psychological

In relation to psychological causes, participants indicated that dementia could be caused by stress from multiple sources, including excessive worrying, a history of trauma, or anger. Social issues, such as domestic violence and financial problems, and mood difficulties, such as hopelessness and despair, were also noted to contribute to dementia. Mistreatment and poor relationships between family members were noted to cause the elderly person to worry, thereby causing stress and leading to dementia.

*“It is poor care. The person might hate herself due to poor care and says that what I’m I doing on earth, majority normally hang themselves (meaning committing suicide) because she may be sick and has no one to take her to the hospital and says that why I’m I living … ”(FGD Female).*

Loneliness and lack of support was identified as a cause of dementia, as was the absence of loved ones who had died or moved away.

*“Losing their [loved ones] for example children and grandchildren causes memory loss”. (IDIMale)*

Additionally, unstable families where an elderly person is left alone, or is not taken for medical care by family members, was the other psychological aspect identified as a cause of dementia.

*“In this community it is because of losing their people for example children and grandchildren also causes memory loss … because you find that he/she stays alone, even children leave him and go far and they do not always come to see her it makes him think a lot and hence leading to memory loss”. (ID17Male)*

#### Nutritional deficiencies and substance use

The majority of the participants from the IDIs attributed dementia to tobacco smoking and alcohol abuse. Some believed that abstaining from alcohol, tobacco, or marijuana could be protective against memory loss.

“*We have a problem of many people who take alcohol in my sub-county: it spoils their brains it causes them memory loss”. (IDI Female)*

Discussions in the FGDs revealed that poor nutrition was also thought to lead to memory loss.

*“If … you don’t give that old woman nutritious food her brain keeps going backwards, [which] causes her memory loss”. (FGD Female)*

*“Food protects against diseases … but sometimes you find that mostly in the villages they feed on one type of food you find that she cooks beans from 1st-31st (meaning the whole month), so it means he doesn’t get the foods which would help in building his memory so feeding also brings forgetfulness or memory loss among the elderly people”. (FGD Male)*

#### Poverty

Participants revealed that when someone is poor, the state of poverty can cause excessive worry which then leads to memory loss. Poverty present throughout life was identified as a cause of dementia, as was loss of previously acquired property or financial resources.

“When *you are poor everything fails, you cannot manage to get anything … the family becomes disorganized you get problems and lack knowledge (meaning memory loss)”. (IDI Female)*

When pressed for the relationship between poverty and dementia, many participants reported that poverty led to excessive worrying and psychological stress, which then resulted in dementia.

#### Social causes

It was noted the traumatic experiences earlier in life could lead to dementia at a later age. Some of the participants reported that childlessness, domestic violence, and unfaithfulness in the family can also cause dementia.

*“ … you may grow up from a bad family with domestic violence … they say that a tree is bent when still young, when it is old it breaks”. (FGD Female)*

#### Traditional beliefs

Traditional beliefs came up among participants as a cause of dementia in elderly people. Participants reported that PWD were attacked by evil spirits or invaded by satanic powers.

*“ … the majority get the diseases of forgetfulness and sometimes they think that they are invaded by Satan … ” (IDI Male).*

One participant attributed the cause of dementia to herbal medicines taken by elderly people.

*“ … culturally some of them, their memories are perturbed by frequent visits to the traditional healers for herbal medicine, when they take them, their way of thinking changes (meaning memory loss)”. (IDI Male)*

### Theme 4: impact of the disease

The impact of the disease was grouped under two sub-themes: (i) Impact on PWD, and (ii) Impact on caregivers.

#### Impact of the disease on PWD

The following categories emerged under the sub-theme of impact of dementia on PWD; (a) Inability to perform personal care (b) Risk of medical and psychological illness, (c) Mistreatment and Risk of Elder Abuse.

#### Inability to perform personal care

Most participants acknowledged that PWD experienced progressive difficulty in performing personal care. This causes PWD to depend on friends and family members for assistance and care.

*“Now there is a person who can no longer take himself to the toilet … some fail to feed themselves...When he gets to that stage, he is unable to do anything for himself but he waits to be helped”. (IDI Male)*

#### Risk of medical and psychological illness

Participants also revealed that PWD were at risk of injuries in attempt to perform personal care. Some of the injuries mentioned included cutting themselves while chopping wood, and or falling into an open fire.

*“ … he may fall in fire and he gets burnt because for such people if you don’t distance them from fire he/she can fall there (meaning in the fire) because he is mentally retarded he can do anything that can even take his life, so personally he encounters such you find that he cuts himself with a hoe or he cuts himself while cutting wood so everything that he uses might harm him because of mental retardation”. (IDI Male)*

Participants also reported that PWD are at a risk of infections because of their behavioral changes including picking up trash, drinking from dirty bottles, and not bathing.

*“ … he may acquire diseases … like when he takes water in those bottles he may get diarrhea, typhoid because he doesn’t know what he is doing”. (IDIFemale)*

*“Those people encounter diseases mostly those related with uncleanness because such people don’t bathe. If she is a woman you know the nature of a woman you find that she acquires some diseases and it disturbs the family”. (FGD Male)*

Participants explained that due to the fact that PWD spend most of their time confined in one place, and may have fecal and urinary incontinence, they are at risk of developing bed sores.

*“ … there are those who fail to get out of the house and he/she keeps sleeping in one place he gets bed sores … ”(IDIMale)*

*“… if you don’t care for such a patient he may rot before he dies, you find that when he defecates, urinates you don’t change for him clothes you find that such things can burn his skin because for him in his mind he doesn’t know what is right … ”. (FGDFemale*)

Participants further reported that PWD faced unpleasant emotional symptoms. These were noted to include expressions of anger, crying, loneliness, regret, misery, self-rejection, hopelessness, and suicidal ideation.

*“The way I know this community, apart from diseases which affect the elderly, they feel unloved because of their age, so the middle class they don’t associate with the elderly, they remain in a small group of the elderly, they don’t access any information from these people, those that I know feel neglected and can never associate with other community members making them lose significance”. (IDI Male)*

*“For me most of the time I always see an old man when he gets disturbed he gets self-rejection and he feels like committing suicide”. (FGD Male)*

#### Mistreatment and elder abuse

Participants revealed that PWD were at risk of mistreatment from others who may think that their behavioral issues were intentional. They reported that some PWD may experience physical abuse by strangers, family, or caregivers; others may be neglected or left alone in the house.

*“The caregivers will not know that he or she has got [memory loss], and they will think that it is intentional so they encounter beatings and failure to clean them”. (IDI Female)*

*“ … others are given goat’s waste that it is millet or peas, or when they know that he or she is elderly, the grandchildren start teasing them, they make him/her eat inedible things”. (IDIMale)*

Some participants identified a risk of abuse of PWD, including rape, physical violence, or homicide. Homicide was identified as a risk from caregivers or family members as well as strangers.

*“ … when he is dependent, he becomes a problem, the bad children go away even some can poison him/her or they intentionally kill them”. (IDI Male)*

*“When she is a woman there are fearless people who rape her, sometimes she dies because they have handled her in a bad way”. (FGD Male)*

Participants noted that PWD are at risk of wandering outside of their village where they can face mistreatment in other communities.

*“ … due to memory loss so you find that he goes to a different village he/she meets people and they beat him or her and he gets bruises by the time you get him you find that the situation is worse”. (FGD Male)*

#### Impact of the disease on the caregiver

Participants perceived a large amount of caregiver stress which they felt was present constantly. The effects of caring for a PWD on caregivers fell under four categories: (a) Financial stress, (b) Social isolation, (c) Handling problematic behaviors, (d) Physical and emotional stress.

#### Financial stress

Financial stress was increased because of caring for a person with dementia due to need for food, medication, and transport costs to the clinic or hospital. The costs of looking after PWD were high and included paying for medical consultations and hospital care in later stages. Participants also reported that financial stress could be caused by the inability to work due to increasing care giving requirements in the home.

*“The caregivers for such people encounter challenges. You may find that a person has his job, for example a government one, but because of the responsibility of taking care of that person he cannot get the time to work and may lose the job and money”. (FGDMale)*

#### Social isolation

Participants identified that when caring for a person with dementia, caregivers were unable to care for themselves including performing personal care, maintaining employment, tending to agriculture, and attending social gatherings.

*“ … it may be difficult for you to go and see your friends or gather with people whether they are far or near … ” (IDI Male)*

*“ … you find that he needs to be cared for and you spend a lot of time on him/her so the caregivers loses on his/her own work and his/her property is lost because he/she spends much of the time taking care of an old man or woman … ”. (FGD Male)*

Responses indicated that a caregiver’s presence maybe constantly required to stop the PWD from escaping, wandering, or spoiling things, which can lead to increased social isolation of the caregiver.

#### Handling problematic behaviors

Participants felt that caring for PWD is challenging especially if they refuse to be assisted with eating or bathing.

*“ … you can cook food and they refuse it or pour it on you, you may wash for him or her, she makes it dirty again … ” (IDI Male)*

#### Physical and emotional stress

Participants reported that caring for PWD was physically tiring and demanding. Additionally, they noted that caring for PWD was emotionally straining and created feelings of mental exhaustion and stress.

*“ … so, when they become elderly, he/she wants you to feed him/her and he/her defecates where he/she is eating from and for you, you don’t want to clean up feces”. (FGD Female)*

*“ … if you are taking care of an elderly person it is very tiring, there are some who are too elderly and cannot move far hence they need support to move them around, such people (meaning elderly) need extra care which you cannot offer to him or her, if the caregiver is not trained he or she may abandon her and look at her as a burden … ”(ID1Male)*

Participants also noted that caregivers sustained a cognitive workload in order to balance multiple competing demands. For example, caregivers need to keep up with many additional details about caring for PWD, in addition to keeping up with multiple other responsibilities.

*“The situation becomes complicated for the caregivers because sometimes you may suffer from such diseases due to overworking using the mind, you get thoughts everything rotates on you because all the time your mind is on the patient and yet you have other responsibilities which you have to fulfill. You have children to take care of, you want to pay school fees, people want food and you have to look for it, electricity, water bills, loans so all those problems are centered on the caregiver … ”. (FGD Female)*

Furthermore, participants reported that if someone was caring for a PWD who had an illness such as tuberculosis or HIV, the caregiver was at risk of contracting the illness.

### Theme 5: views on how to address unmet needs in dementia care

Participants expressed different views regarding what should be done to help PWD and their families. Participants’ views fell into categories of emotional support, socioeconomic support, and cultural sensitization.

#### Emotional support

The need for emotional support for both caregivers and PWD was strongly mentioned by respondents. Identified forms of emotional support included having someone available to talk with, showing empathy, practicing effective communication, and providing counseling. It was noted that PWD could benefit significantly from caregivers and community members who were attentive and loving, who visited them, and who did not blame them for their disease.

*“ … so for you who is taking care, you should not talk to her rudely, you need to comfort her so that she feels happy … when you are near her you call her ‘mummy’ and counsel her, you can even say my child even feed her, don’t you know that you fed me when I was a child so you are also my child she feels happy.”(FGD Female)*

Participants felt it essential to have external sources of emotional support as a way to address the problem of dementia.

*“For me it would be counseling or get people not within the community to comfort and counsel [PWD] … there should be an organization which should always visit, counsel, and comfort and give them hope to live and even offer medical treatment. They should support their families in taking care of them”. (IDI Male)*

#### Socio-economic support and services

In most communities, PWD depended upon family members and communities for social and financial support. Participants revealed that costs of transportation to health facilities were problematic. Some participants suggested that the government should set up facilities to care for elderly people.

*“What would help them we do not have it in Africa, it is taking them to the elderly homes and treat them from there, but it is difficult to take a person from his or her home, or away from relatives but it would be possible mostly for those without enough care, they should put them in one place and care for them from there.” (IDI Male)*

Other participants noted that homes for elderly people did exist in Africa, but were not available to the public. Participants further emphasized that the established elderly facilities should be equipped with trained caregivers.

*“ … when people become elderly the government should plan for them to get care from trained people because though the elderly have caregivers, he/she doesn’t know what to do … ”(IDIMale)*

One participant urged that there should be a representative for the elderly at every level of the governing councils to become a voice for elderly people.

*“I would advise that on the local council committee there should be a person to represent the elderly people. For example, someone who represents health in particular for [PWD] … they should have that person who has a good memory but that person should think for such people”. (IDIMale)*

A few of the participants suggested that the government should bring services to diagnose and treat dementia closer to local communities to increase access to dementia care.

*“ … another thing is to see that at the health facilities the medicine and care for the elderly are availed they make sure that they treat them because there is when they reach at the health facility … ”(FGD Male)*

Other participants noted that the government should provide medical supplies to support caregivers for patients who were cared for in the home.

*“For example, the elderly person who is HIV positive you find that the person who is taking care of him/while bathing him he/she would need to use gloves may be freely provided by the government because there should be an arrangement for caring for the elderly by the government”. (FGD Male)*

Other participants believed PWD could be delivered and healed through prayers. These participants tended to believe that in some people dementia was due to satanic powers and witchcraft. They felt that PWD should seek help from witch doctors or go to churches for prayers, deliverance, and preaching.

*“Some are bewitched so when they become like that, we take them to churches to pray for them and they get better. We pray for him/her and he becomes fine, there are devils which can make a person like that but after praying it goes [away]”. (IDI Female)*

One participant felt that to help manage the challenge of dementia, one ought to first pray for God to give them strength and courage to take up the role of caring for a person with dementia.

*“ … most times to do that job I ask God to give me love, energy, and capacity because if you don’t pray hard it fails you”. (FGD Female)*

One participant emphasized that elderly PWD need outing activities to help them relax themselves.

*“ … like my children who are able should take me out I take some tea and sodas, while listening to those good songs/music and they take me back home because I don’t have any other chance of taking myself”. (IDI Male)*

#### Sensitization

Creating awareness to improve knowledge and understanding of dementia among communities was deemed vital by participants.

*“ … I think the community should be sensitized to think about these people so that when he or she becomes elderly and gets memory loss we should not refer to them as mentally disoriented. They are like other people and they need help”. (IDI Male)*

Participants noted that there were multiple non-governmental resources which could provide training, counseling, and assistance to caregivers of PWD, including religious leaders and Village Health Teams (a group of individuals providing healthcare support at a village level).

Participants believed that counseling on how to manage and care for PWD would help address the problem of dementia. Conducting research to find more about dementia and address its challenges was also recognized to be valuable.

*“There should be people to identify that this is in existence and it is a problem in our society … [there] should be people to conduct research from the grass root level”. (IDIMale)*

## Discussion

This qualitative study focused on community perceptions of PWD and their caregivers in rural southwestern Uganda. The most common label used to describe PWD was “okuhuga,” which translates as “mental disorientation.” Descriptions of the symptoms of dementia matched closely with other communities in Sub-Saharan Africa [10]. Many participants accepted dementia as a normal consequence of aging, but medical and social explanations were also described. Our results demonstrate the significant burden of dementia on PWD and their caregivers. Effects on caregivers included economic constraints, physical demands, emotional strain, and social isolation. Overall, our findings support a strong need for community-based education and greater infrastructure including mental health providers, caregiver supports, and governmental aid for PWD and caregivers in local communities in southwestern Uganda.

Our results show a link between dementia and “mental retardation.” This mirrors results from a study in Nigeria that included descriptions of dementia such as “disease of insanity” and showed that 36% of respondents associated dementia with shame and embarrassment [17]. Dementia-related stigma is common worldwide, and impedes help-seeking behavior of PWD and caregivers [18]. While our study did not specifically examine stigma, further investigation is needed to fully understand the amount of stigma for PWD in this population. Efforts to reduce stigma may need to be integrated into any future interventions to improve care for PWD in southwestern Uganda.

Knowledge about the causes of dementia was described by participants using both a biomedical and a social model. Many participants reported that dementia was a natural part of the aging process, and did not perceive dementia to be a medical problem. This replicates findings from studies in Europe, the US, East Asia, Israel, and Australia, which show that nearly half of the general public believes that dementia is a normal consequence of aging [19]. Other participants reported that dementia may be caused by untreated medical conditions such as nutritional deficiencies and syphilis, or by side effects from allopathic medications or herbal treatments. These explanations were found to encompass some, but not all, of the current medical understanding of dementia. Still others attributed dementia to satanic powers, witchcraft, or life stress. This range of responses about the causes of dementia mirrors findings from a similar study in Tanzania [10]. Overall, our findings show partial knowledge about the risk factors and causes of dementia, and highlight the need for increased community-based education.

Participants reported that the primary responsibility for caring for PWD fell on local caregivers, and was associated with significant physical, emotional, and financial strain. Participants revealed that caregivers often had to forego leisure activities or leave work to take care of the PWD. Caregiving was reported to cause social isolation, anger, irritability, fatigue, and suicidal ideation. Prior studies have also shown a high risk of depressive symptoms [20] and social isolation [21, 22] in caregivers of PWD. Notably, participants in the present study did not identify any positive aspects of caregiving such as companionship, self-esteem, personal growth, or the satisfaction of helping others, which have been reported in the literature [23, 24]. Since we did not ask specifically about positive aspects of care giving, this could partially represent ascertainment bias. However, it also likely reflects high levels of caregiver stress in our population, which had high levels of caregiver burden risk factors including depression, social isolation, low socio-economic status, and lack of choice in being a caregiver [20, 21]. In other settings, caregiver support groups have proved to be effective in enhancing caregiver quality of life [25], and should be included as part of any future interventions to improve dementia care in local communities [26]. Additionally, literature shows that the behavioral and psychological symptoms of dementia, such as aggression, wandering, hallucinations, delusions, are strongly predictive of caregiver burden [27]. Medical interventions to reduce behavioral symptoms in PWD also significantly improve caregiver mood, coping skills, and self-efficacy [21], and should be integrated with other focused interventions. Given the reliance of PWD on community-based caregivers in our population, partnering with local caregivers will be paramount to improving the well-being of PWD in rural Uganda.

Collaborating with local organizations will be necessary in order to implement formal services for PWD and caregivers. Even in settings with greater access to services, caregivers may be reluctant to participate due to a lack of awareness or perceived lack of need [28]. A study in Australia found that culturally and linguistically diverse communities may be unfamiliar with the idea of formal dementia services, and may hold strong cultural beliefs about the value of family-based caregiving [29]. Our participants reported that non-governmental organizations such as churches and traditional healers are already involved in the care of older adults and PWD in southwestern Uganda. Similar findings have been described in Tanzania, where both traditional healers and Christian faith healers were described as resources for PWD to seek help and treatment using prayers, plants, and witchcraft [30]. Therefore, religious leaders and traditional healers may provide additional avenues to link PWD with medical care and offer advice and emotional support to caregivers [10].

Our study supports a need for increased governmental services for PWD. Participants reported that the government could play a role in the care of PWD through the establishment of elderly homes, thereby alleviating caregiver stress and allowing caretakers to continue to continue employment. They also mentioned that representation of elderly leaders in local governments would help to improve the conditions of PWD and their caregivers. Taken together, our results echo guidelines put forth by the World Health Organization and Alzheimer’s Disease International, which advocate for improving access to early dementia diagnosis, increasing public awareness, reducing stigma, and providing caregiver supports [2]. Implementing these strategies for PWD and caregivers represents an opportunity to foster economic development by employing of local mental health workers, improving economic productivity of caregivers, and decreasing the need for PWD to enter high-cost care settings. However, in resource-limited countries like Uganda which have seen a significant growth in the aging population, substantial pre-planned efforts will be needed at both national and community levels in order to implement these strategies. For example, efforts to improve care for PWD must involve strengthening the current public health care system to empower local providers to manage chronic conditions in older adults, including medical comorbidities, mental health issues, and dementia.

One limitation of our qualitative study design is the inability to draw statistical conclusions about the prevalence of different beliefs about dementia in local communities. Therefore, this study should be followed by future quantitative and interventional studies. Second, our study design focused on rural communities in southwestern Uganda, in a limited geographical area. This design was intended to capture the local perceptions about PWD, and to inform future interventions seeking to improve care for PWD. Since our study replicated similar findings about dementia perception in other regions in Sub-Saharan Africa, it is likely that our findings are largely translatable to similar regions. Finally, our study did not capture perceptions about PWD in urban communities, which may have easier access to medical resources and information about dementia. Future studies are needed in urban centers in Sub-Saharan Africa, since urban communities may require different types of resources and infrastructure to treat PWD.

## Conclusions

This study revealed community perceptions of PWD and caregivers in southwestern Uganda, and highlighted the need for improved care and support of PWD and their caregivers. Collaborating with local caregivers to reduce levels of caregiver burden can also improve wellbeing of PWD. Communities require increased aid from both non-governmental and governmental organizations in order to access much-needed emotional support and education for caregivers of PWD. Increased representation of older adults in local government can help advocate for the needs of PWD. Further development of integrated mental health care for elderly people is needed to improve management and care of dementia.

## Data Availability

The full dataset generated and analyzed during the current study are not publicly available in order to maintain the privacy of the individuals interviewed during this study. De-identified data can be made available from the corresponding author on reasonable request.

## Abbreviations

ADLs: Activities of daily living
PWD: People with dementia
FGDs: Focus Group Discussions
LMIC: Low and Middle Income Countries
LCs: Local Councils
CDOs: Community Development Officers
HIV: Human Immuno-Deficiency Virus
